# Supplementary material for: Effects of different drying methods on smears of canine blood and effusion fluid
Source: PeerJ. 2020 Oct 27;8:e10092. doi: 10.7717/peerj.10092 (PMC7597622; doi:10.7717/peerj.10092)
Supplement: Supplemental Information 1 [file peerj-08-10092-s001.docx]

Supplemental table: Characteristics of effusion fluids.

| **Sample type** | **TNCC/µL** | **RBC/µL** | **Protein (g/dL)** | **Cytologic interpretation** |
| --- | --- | --- | --- | --- |
| Peritoneal effusion | 2,940 | 10,000 | < 2.5 | Mild neutrophilic inflammation |
| Peritoneal effusion | 680 | 60,000 | 3.7 | High-protein transudate, lymphocyte-rich |
| Peritoneal effusion | clotted | clotted | < 2.5 | Mild to moderate neutrophilic inflammation |
| Peritoneal effusion | clotted | clotted | < 2.5 | Low-protein transudate |
| Pleural effusion | 3,040 | 10,000 | 3.3 | High-protein transudate, lymphocyte-rich |
| Pleural effusion | 13,180 | 70,000 | not performed (lipemic) | Neutrophilic inflammation; presumptive chylous effusion |
